# Thermal-Induced Alterations in Phenolic and Volatile Profiles of Monovarietal Extra Virgin Olive Oils

**DOI:** 10.3390/foods13213525

**Published:** 2024-11-04

**Authors:** Dora Klisović, Anja Novoselić, Marina Lukić, Klara Kraljić, Karolina Brkić Bubola

**Affiliations:** 1 Institute of Agriculture and Tourism, K. Huguesa 8, HR-52440 Poreč, Croatia; klisovic.d@gmail.com (D.K.); novoselic.anja@gmail.com (A.N.); marina@iptpo.hr (M.L.); 2 Faculty of Food Technology and Biotechnology, University of Zagreb, Pierottijeva 6, HR-10000 Zagreb, Croatia; kkraljic@pbf.hr

**Keywords:** thermal treatment, monovarietal extra virgin olive oil, oxidative stability, PCA, phenolic compounds, volatile compounds

## Abstract

In the present study, the influence of heating on the evolution of oxidative indices, antioxidant activity, phenolic and volatile compounds in monovarietal extra virgin olive oils (EVOOs) obtained from Leccino, *Istarska bjelica*, and Buža cultivars was investigated. The samples were submitted to heating in an air oven (180 °C and 220 °C), simulating usual roasting conditions typical for Mediterranean cuisine. The decreases in the oxidative indicators, phenolic and volatile compounds were more pronounced at higher heating temperatures, underlining the temperature dependency of the oxidative degradation during heating conditions. Despite this, it must be emphasized that a significant amount of phenolic compounds and antioxidative activity remained preserved after the heating treatment. Each oil cultivar showed some specificity during the course of the thermal degradation. Hydroxytyrosol acetate among phenolic compounds and octanal, (*E*)-2-octenal, hexanal, 3-pentanone, and 1-penten-3-one among the volatiles were underlined as possible markers of thermal oxidation. Principal component analysis revealed that the content of volatile compounds in monovarietal EVOO samples distinguished samples primarily by the heating temperature, while the changes in the phenolic compounds were cultivar-dependent aside from being influenced by the temperature of heating.

## 1. Introduction

The Mediterranean diet is characterized by the daily consumption of extra virgin olive oil (EVOO) as basically the exclusive source of dietary lipids [1]. The health benefits of this diet have been associated with the consumption of EVOO. Besides using it fresh, it is highly recommended to use EVOO as a cooking oil to prepare meals (e.g., meat, fish, and vegetables). Currently, EVOO is considered as the most suitable vegetable oil for cooking primarily due to its fatty acid content high in monounsaturated fatty acids, being more resistant to oxidation compared to polyunsaturated fatty acids [2]. According to a study reported by Guillaume et al. [3], EVOO demonstrated the highest stability when heated, closely followed by coconut oil, avocado oil and high-oleic acid seed oils. In contrast, seed oils such as canola, grapeseed, sunflower, and rice bran oils exhibited lower oxidative stability due to their higher polyunsaturated fatty acid content and lower levels of natural antioxidants [3].

The fatty acids gathered in triglycerides present virgin olive oil’s major fraction (~98% of the total content), whereas the minor fraction (~2%) consists of numerous compounds such as phenolic and volatile compounds, tocopherols, terpenes, and squalene that contribute to its high nutritional and functional value [4]. Among these, phenolic compounds are especially valued for their antioxidant properties, playing a critical role in oxidative stability under high temperatures [5].

During heating, the chemistry of lipid oxidation is complex since thermal and oxidative reactions occur simultaneously [5,6]. The mechanism of thermal oxidation is similar to autoxidation but initiated by heat and occurring at a higher rate [5,7]. The oil undergoes several chemical reactions that influence its chemical composition, oxidative stability and aroma quality [7]. The impact of cooking method on the composition of EVOO varies depending on the techniques used [2]. Despite common use of EVOO in cooking, existing research on stability of volatile and phenolic compounds during heating has notable areas that should be further explored. In the available literature, there can be found several studies that investigated the influence of thermal treatment on the EVOO fatty acid composition and phenolic compounds [8,9,10,11,12,13,14,15], while only a few of them considered the composition of volatile compounds which is directly related to the unique aroma of EVOOs [16,17]. However, most of these studies focused on the investigation of the rate of oil oxidation during prolonged heating duration for several hours at a single temperature [8,9,10,11], whereas only a minor number investigated the influence of the different heating temperature [12,17,18,19]. Similar issues in the investigation of other vegetable oils regarding the duration of heating have been reported by Mitrea et al. [20] and they proposed an investigation of short-time heating in order to mimic realistic domestic cooking conditions. They have found that short-time exposure at a high temperature (30 min at 180 °C) also affected negatively the physicochemical properties of other vegetable oils such as sunflower, rapeseed, maize, palm, and coconut oils [20]. Moreover, most of these studies fail to consider realistic domestic cooking conditions, like roasting, where the oil is highly susceptible to oxidation due to large surfaces exposed to hot air or consider only one monovarietal olive oil during thermal treatment [19].

Among several others [21], most recently, the EVOO cultivar has been underlined as the most important factor indicating the oxidative stability of the oil [22]. Therefore, the effect of cultivar-specific phenolic profiles on EVOO’s oxidative stability under real domestic heating conditions needs to be further explored. Also considering that the temperature of heating was mainly responsible for the polyphenol depletion in the Hojiblanca cultivar of EVOO during sautéing with no significant effects of heating time [12], it is of high importance to investigate this matter profoundly in different monovarietal oils with a specific phenolic profile. Moreover, changes in less investigated chemical compounds such as volatiles under heating conditions need to be considered in further studies.

Due to all that has been stated, this study aimed to investigate the influence of heating at two different temperatures (180 °C and 220 °C) on the oxidative stability of different monovarietal EVOOs by determining changes in its quality parameters related to the oil oxidation status, antioxidant activity, and the composition of phenolic and volatile compounds. The thermal treatment included heating in an air oven at two different temperatures, simulating usual roasting temperature conditions typical for the Mediterranean cuisine. The hypothesis was that the heating under realistic domestic conditions will significantly alter EVOO composition, particularly affecting phenolic and volatile profiles, and that these changes would vary among monovarietal oils due to cultivar-specific composition. To explore this hypothesis, three monovarietal olive oils obtained from Croatian autochthonous cultivars *Istarska bjelica* and Buža and an introduced cultivar Leccino were selected as the most common cultivars in the Istrian region of Croatia and known for their unique phenolic profiles and sensory attributes [23]. Using these monovarietal oils, we sought to assess not only the impact of temperature on oxidative stability and compositional changes but also to elucidate the role of their initial chemical composition as influenced by cultivar. To our knowledge, this study is among first to analyze these monovarietal EVOOs under real domestic heating conditions. The results obtained from this study will enhance the understanding of how different high-temperature exposures affect the preservation and transformation of beneficial and flavor-related compounds in EVOO, while also highlighting the role of specific cultivars under simulated domestic heating conditions.

## 2. Materials and Methods

### 2.1. Oil Sample Preparation

Olive (*Olea europaea* L.) fruits of Leccino (L), *Istarska bjelica* (IB), and Buža (B) cultivars were manually harvested on October 10, 2018, from the same orchard (Istria region, Croatia). The fruits were in the ripening stage most common for the harvest of each olive cultivar in Croatia. The ripening index was 2.76, 1.10, and 1.53 for L, IB, and B cultivars, respectively, determined by the method of Beltrán et al. [24]. Monovarietal EVOO samples were obtained within 24 h from harvest using a two-phase extraction plant (Model SPI 222 S, Pieralisi, Iesi, Italy) and filtered using a filter press system (OV110, Omniafiltra, Alife, Italy). Until the start of the experiment, the oils were kept in 1 L dark glass bottles, filled to the top.

For the experiment, 50 g of each monovarietal EVOO was placed in a 9 × 7 × 7 cm opened Pyrex glass roasting dish. Afterward, samples were heated for one hour in an air oven at 180 °C (± 4 °C) and at 220 °C (± 4 °C). After heating, the oil samples were left to cool down to room temperature (approximately for 30 min) and analyzed immediately after, starting with quality parameter analyses. All heating treatments were performed in triplicates. The unheated fresh oil served as the control oil. During the cooling period of the thermally treated oil samples, the control oil (in triplicate) was also left in an open glass dish at room temperature (22 ± 2 °C) for 30 min prior to analysis.

### 2.2. Quality Parameter Determination

Quality parameters related to the oil oxidation status, peroxide value (PV), and spectrophotometric indices (K_232_, K_268_, and ΔK) in oil samples, before and after heating treatment, were determined according to standard analytical methods [25]. The extinction coefficients, K_232_ and K_268_, were determined by measuring the absorption at 268 and 232 nm, respectively, on a Varian Carry 50 spectrophotometer (Varian Inc., Mulgrave, Victoria, Australia). The value of ΔK was obtained following the formula ΔK = K_268_ − [(K_264_ + K_272_)/2].

### 2.3. Phenolic Compounds Analysis

The extraction and analysis of phenolic compounds in oils was performed according to Jerman Klen et al.’s study [26] and a later modification reported by Lukić et al. [27] on an Agilent Infinity 1260 system (Agilent Technologies, Santa Clara, CA, USA). A Kinetex PFP column (100 mm length × 4.6 mm i.d., 2.6 µm particle size) equipped with a guard (2.1 mm length × 4.6 mm i.d.) was utilized (both Phenomenex, Sydney, Australia). A previously described 20-step gradient run reported by Lukić et al. [27] was employed for the analysis.

Phenolic compounds were identified by comparing their retention times and UV/Vis spectra to those of pure standards as well as from the literature [26]. Detection wavelengths were set to 280 nm for simple phenols, lignans, secoiridoids, and vanillic acid, 320 nm for vanillin and p-coumaric acid, and 365 nm for flavonoids.

Standard calibration curves of the available phenolic compound were used for quantification, while for the quantification of hydroxytyrosol acetate, acetoxypinoresinol, and secoiridoids concentrations, a semi-quantitative approach was employed and their concentrations were expressed as hydroxytyrosol, pinoresinol, and oleuropein, respectively, assuming a response factor equal to one [27]. Total identified phenolic compounds (TIPCs) presented the concentration sum of all identified phenolic compounds determined by the HPLC method used.

### 2.4. Radical Scavenging Activity

The antioxidant activity of the olive oils was assessed by measuring their free radical-scavenging capacity using the DPPH radical method, as reported by Koprivnjak et al. [28]. Absorbance readings were taken with a Varian Cary 50 spectrophotometer (Varian Inc., Mulgrave, Victoria, Australia). Calibration curves were generated using Trolox solutions of known concentrations for accurate quantification.

### 2.5. Volatile Compound Analysis

Volatile compounds were extracted using headspace solid-phase microextraction (HS-SPME) and analyzed applying a method based on Brkić Bubola et al. [29] and updated in Brkić Bubola et al. [30]. A 1 cm divinylbenzene/carboxen/polydimethylsiloxane (DVB/CAR/PDMS) SPME fiber (50/30 μm f.t., Supelco, Bellefonte, PA, USA) was used for extraction. The analysis took place on a Varian 3350 gas chromatograph (Varian Inc., Harbor City, CA, USA) equipped with a split/splitless injector set to 245 °C, a flame ionization detector (FID) at 248 °C, and an Rtx-WAX capillary column (60 m × 0.25 mm i.d. × 0.25 μm f.t.; Restek, Bellefonte, PA, USA). The oven temperature was programmed to start at 40 °C, increase to 170 °C at 2.5 °C/min, then to 245 °C at 15 °C/min, and holding at 245 °C for 15 min, with helium as the carrier gas.

The identification of volatile compounds was carried out using a Varian 3900 GC coupled to a Varian Saturn 2100 T ion trap mass spectrometer (Varian Inc., Crawley, the UK) by comparing retention times and mass spectra with those of pure standards and the NIST05 library. Quantification relied on calibration curves of pure standards [29,30]. Since (*Z*)-2-penten-1-ol and (*Z*)-3-hexenyl acetate had an equal retention time, their concentrations were expressed as the corresponding sums. The volatile compounds without available standards (*Z*-2-hexenal, *E*-3-hexenal, and *Z*-3-hexenal) were quantified semi-quantitatively, and their concentrations were expressed as equivalents of *E*-2-hexenal, assuming a response factor of one [29,30]. Total volatiles were reported as the concentration sum of all identified volatile compounds.

### 2.6. Statistical Analysis

Differences among samples were assessed for statistical significance using one-way ANOVA, followed by Tukey’s honest post hoc multiple comparison test to evaluate mean differences (*n* = 3) at a significance level of *p* < 0.05. Principal component analysis (PCA) was conducted on group samples based on the heating treatment and cultivar. Statistical analyses were carried out using Statistica software, version 13.2 (StatSoft Inc., Tulsa, OK, the USA).

## 3. Results and Discussion

### 3.1. Quality Parameters During Heating

Quality parameters (PV, K_232_, K_268_ and ΔK) provide information regarding the oxidation status of the olive oil as standard indicators of quality regulated by the European Commission [25]. However, they might also be used as oxidation indicators in oils subjected to elevated temperatures during cooking [9,11]. The results of the quality parameter analysis of unheated and heated monovarietal oils are given in Table 1. All the unheated oil samples where within the prescribed limits corresponding to the EVOO category [25].

The obtained results showed that the levels of PV after the heating treatment where not clearly related to the heating temperature (Table 1). Precisely, after heating at 180 °C and 220 °C, the PV significantly increased in the L cultivar EVOO without surpassing the “extra” category [25]. Otherwise, in IB and B EVOO, after the heating treatment, the PV was unaffected or even decreased compared to unheated samples, whereas a lower PV was observed at 180 °C compared to 220 °C (Table 1). A decrease during the first hour of heating commercial EVOO (initial PV 10.54 meq O2/kg) at 180 °C was previously also observed by Carrasco-Pancorbo et al. [10], which correlates with results for the IB and B EVOOs obtained in this study (Table 1). A lower level of hydroperoxides in the virgin olive oils (VOOs) from Arbequina and Picual cultivar heated in an air oven at 180 °C after two hours of heating compared to the unheated oil was also observed by Allouche et al. [9]. Lower PVs after heating were expected, considering that the PV provides information about the current state of primary oxidation products, corresponding to the level of hydroperoxides, and having in mind that the transformation of hydroperoxides into secondary products (e.g., aldehydes and ketones) is faster at elevated temperatures [17]. Due to the known high reactivity of unstable hydroperoxides, it might be hypothesized that at this particular point of the analysis, when no changes in the PV were observed, the formation and decomposition rate of hydroperoxides was similar [31].

The extinction coefficients, K_232_ and K_268_, show strong correlation with the applied temperature of heating (Table 1). The most notable increases were observed in oils heated at 220 °C, where K_232_, K_268_, and even ΔK, increased above the prescribed legal limits for VOO quality [25], indicating accelerated oxidative degradation at higher temperatures (Table 1). All the oil samples heated at 180 °C showed major increases in K_268_, surpassing the corresponding legal limits for EVOO and VOO quality [25], whereas the K_232_ oxidation indicator remained unchanged (Table 1). Observed changes indicate a high presence of both primary and secondary oxidation products after oil heating, including the increase in conjugated trienes and carbonyl compounds, even at lower heating temperatures. In all the oils heated at 220 °C, both K_232_ and K_268_ and even ΔK increased above the prescribed legal limits for VOO quality [25], indicating accelerated oxidative degradation at higher temperatures. Similar findings were reported in a previous study where both K_232_ and K_268_ increased above the limits for EVOO after only 10 min of heating at 180 °C and 220 °C [18].

### 3.2. Phenolic Compounds and Radical Scavenging Activity

**Figure 1:**
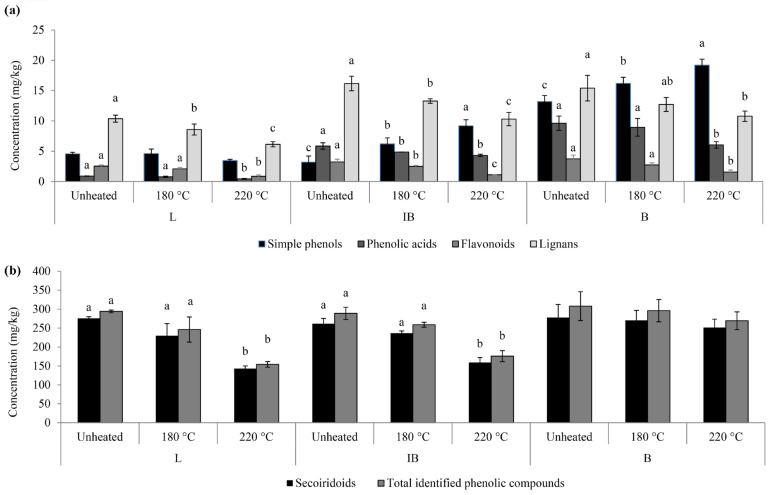
Groups of phenolic compounds in monovarietal olive oil samples unheated and subjected to 1 h of heating at 180 and 220 °C. Data are expressed as means ± standard deviation (*n* = 3), calculated as a sum of individual phenolic compounds: (**a**) simple phenols, phenolic acids, flavonoids, lignans, and (**b**) secoiridoids, and total identified phenolic compounds. Columns labeled with a different letter within the same group of phenols and single monovarietal olive oil (Leccino—L, *Istarska bjelica*—IB and Buža—B) at different temperature of heating are statistically different (Tukey’s test, *p* ˂ 0.05).

The unheated monovarietal EVOO samples contained a similar TIPC, ranging between 280 and 310 mg/kg (Figure 1b).

**Figure 2:**
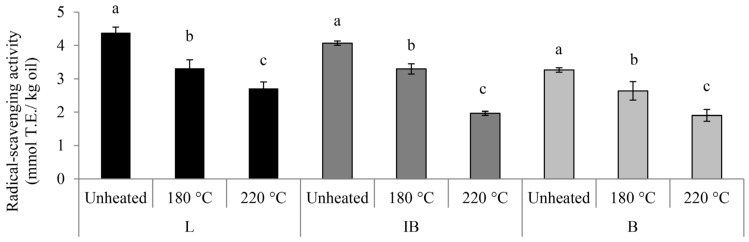
Radical scavenging activity in monovarietal olive oil samples unheated and subjected to 1 h of heating at 180 and 220 °C. Data are expressed as means ± standard deviation (*n* = 3). Columns labeled with a different letter within the single oil cultivar (Leccino—L, *Istarska bjelica*—IB and Buža—B) at different heating treatments are statistically different (Tukey’s test, *p* ˂ 0.05).

Correspondingly, the initial levels of the radical scavenging activity (RSA) in the EVOOs were similar, ranging from 3.3 to 4.4 mmol T.E/kg (Figure 2).

Oleocanthal, oleacein, and oleuropein aglycone isomer I were the most abundant compounds that differed in concentration between investigated monovarietal oils (Table 2). B EVOO was characterized by a high initial content of oleuropein + ligostroside aglycones I & II and hydroxytyrosol acetate, whereas IB EVOO was characterized by a high initial content of oleuropein aglycone I, which corresponds to the findings of Lukić et al. [23], where these compounds were attributed as reliable markers for the differentiation of IB and B EVOO. L EVOO was characterized by having the highest content of oleacein (Table 2). Genetics might be the primary source of such large cultivar diversity in the phenolic profile, as highlighted in a study in which thirty Greek monovarietal olive oils obtained from different cultivars were considered [32].

During heating, phenolic compounds are expected to degrade due to their antioxidant role in the delay of the lipid oxidation [12,33]. After heating at 180 °C, the TIPC in all the monovarietal EVOOs remained unchanged (Figure 1b). However, this was not the case in the concentration of individual phenolic compounds where some significant changes were observed after heating at 180 °C (Table 2). Among secoiridoids, the most abundant group of phenols, oleuropein and its aglycones decreased significantly. The most pronounced decrease was noted for oleuropein isomer I, which decreased by 65%, 65%, and 67% in L, IB, and B oils, respectively (Table 2). The observed result was not unexpected, considering that the hydroxytyrosol derivatives are more susceptible to degradation at high temperatures compared to tyrosol derivatives due to their greater reactivity deriving from their additional hydroxyl group [8,12]. Differently, the concentration of oleacein and oleocanthal slightly increased in both IB and B oils after heating at 180 °C (Table 2). The possible increase in oleocanthal was even more pronounced in B oils after heating at 220 °C, whereas in L oils, it remained unchanged compared to the initial concentration (Table 2).

The higher heating temperature of 220 °C influenced a significant degradation in the total identified phenolic content of L and IB EVOOs by around 47% and 39%, respectively, while remaining statistically unchanged in B EVOO (Figure 1b). The TIPC in B oil remained mostly unchanged due to the increase in oleocanthal concentration after the heating treatment (Table 2).

The increase correlated with the previously reported minimal loss of oleocanthal concentration of 16% after heating at 240 °C for 90 min [34]. Such a strong heat resistance of oleocanthal was also reported for shorter heating periods up to ten minutes [18]. It might be that a high level of oleocanthalic acid or another oxidized form of oleocanthal is formed in B oil after heating due to the oxidation of oleocanthal [35] that is not identified with the HPLC method used, influencing the apparent increase in the oleocanthal concentration (Table 2). Regardless of the increase, it might be that its biological activity was reduced without being in direct correlation with its concentration [34]. The results from the radical scavenging activity (RSA) contribute to the stated information due to the fact that the RSA in the B oil was decreased by the heating treatment (Figure 2) despite the preservation of the TIPC.

Minor groups of phenolic compounds, phenolic acids, flavonoids, and lignans, have shown a higher decrease rate with the rise in the heating temperature (Figure 1a). Lozano-Castellón et al. [19] have also found that these minor group of phenolic compounds were the most degraded compounds in Picual EVOO during oven heating, deep-frying, and sautéing. Among them, lignan pinoresinol remained resistant during heating in all of the investigated monovarietal oils (Table 2), which was in accordance with the results for Hojiblanca EVOO, where slight increases with increased time and temperature were reported in pinoresinol concentration [12]. An increase in the concentration of simple phenol hydroxytyrosol acetate was noted in all the monovarietal oils after heating at 220 °C (Table 2). Usually, during heating, the hydrolysis of the secoiridiods aglycones gives rise to the increase in simple phenols such as tyrosol and hydroxytyrosol [36]. It might be that the noted increases in hydroxytyrosol acetate concentration also originate from the decomposition of secoiridoid aglycones due to a higher temperature of heating (220 °C). Therefore, its increase in concentration could indicate higher temperatures of heating and serve as thermal oxidation markers among phenolic compounds.

Higher temperatures of heating accelerated the thermal oxidation in oils, already indicated by the quality parameter analysis (Table 1) and confirmed in other studies [17]. This correlates with the trend observed for the RSA results, where a notable decrease was detected already after oil heating at 180 °C and was even more pronounced after 220 °C in all the samples (Figure 2). The phenolic compounds act as antioxidants while being decomposed during heating this way, preventing the onset of the oxidation of the fatty acids and other compounds and maintaining the oxidative stability in the oils [2,5]. Considering that the initial TIPC was comparable among monovarietal oils (Figure 1b), the perceived discrepancy of the phenolic content development after the heating treatment indicates the direct influence of the specific chemical composition of the initial unheated oil cultivar. The changes in phenolic compounds after heating were already described as cultivar-dependent and also its thermal properties as strongly influenced by the oil chemical composition [9]. The stated results will be further discussed with the PCA analysis in Section 3.4.

### 3.3. Volatile Compounds

The initial volatile profile of unheated monovarietal EVOOs was characterized by the predominant content of (*E*)-2-hexenal (Table 3), most relevant for its green flavor attributes and typical for high-quality EVOOs [23]. After the heating treatment at 180 °C, (*E*)-2-hexenal was almost completely reduced by 94%, 92%, and 95% in L, IB, and B oils, respectively. The higher heating temperature influenced an exceeded rate of thermal oxidation, due to which the almost complete degradation of (*E*)-2-hexenal was detected (Table 3). This corresponds with the study of Giuffrè et al. [17] where, after the heating treatment (220 °C, 120 min), only 1.7% of (*E*)-2-hexenal was preserved in the EVOO produced in the Calabrian region of Italy. A similar trend was noted for most of other detected volatile compounds as regards the extensive losses provoked by the increased temperature of heating (Table 3). Mostly, these compounds are lost due to their volatilization influenced by high temperatures [37].

Despite the prevalent loss of volatile compounds, some volatile aldehydes and ketones increased in content (Table 3), presumably by being less volatile and produced from the oxidation of the fatty acids [2]. It could be that such compounds are firstly lost due to their volatilization and subsequently formatted during the oxidation of the unsaturated fatty acids [38]. The C5 group of volatiles increased in content in all the heated oil samples due to the significant increase in 3-pentanone and 1-penten-3-one concentration (Table 3). In addition, a significant increase in the hexanal concentration, the second most abundant volatile compound, was noted (Table 3). This coincides with the previously acknowledged role of hexanal as a good indicator of lipid oxidation during heating conditions [38]. Hexanal, a C6 aldehyde, derives from the oxidation of linoleic fatty acid [17,38], which is supported by the results of the fatty acid analysis, where a slight decrease in linoleic fatty acid is noted (Table 2). Among other compounds, octanal and (*E*)-2-octenal increased in concentration at both heating temperatures. The increases were greater with the rise of the heating temperatures in all the mentioned compounds (Table 3). This correlates with the increase in (*E*)-2-octenal concentration detected in EVOO after heating at 180 °C and 220 °C for 60 min [17]. However, in the same study, octanal decreased during heating [17], which was contradictory to the results obtained in our study (Table 3), which indicated the possible role of the specific cultivar in the formation of volatiles during heating. The extensive formation of secondary oxidation products, such as aldehydes was already indicated by the increased K_268_ value (Table 1), which corresponded with the described development of volatile aldehydes (Table 3). Both unsaturated (*E*)-2-octenal and saturated aldehydes (hexanal and octanal) have a role in the aroma of the oil by contributing to the rancid and fatty negative sensory attributes [39], which is the typical aroma of heated oils.

### 3.4. Principal Component Analysis

**Figure 3:**
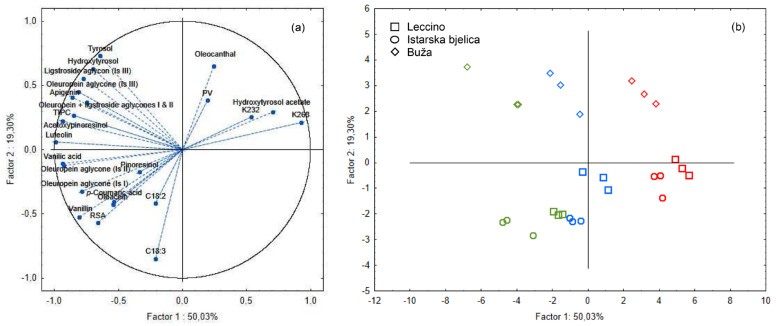
Separation of olive oil samples according to olive variety along principal components PC1 and PC2: (**a**) Loading plot of the selected variables (PV—peroxide value; K_232_, K_268_—spectrophotometric indices; fatty acids C18:2 and C18:3; and phenolic compounds) of the first two factors (PC1 and PC2). (**b**) Score plot projecting the monovarietal virgin olive oils Leccino (□), *Istarska bjelica* (ᴑ) and Buža (◊); unheated (green) and heated in an air oven for 1 h at 180 °C (blue) and 220 °C (red).

**Figure 4:**
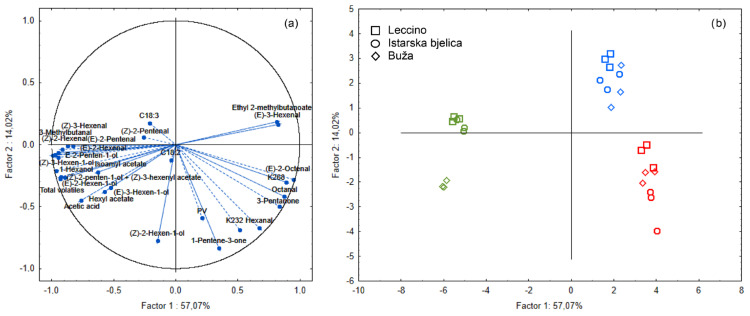
Separation of olive oil samples according to heating treatment along principal components PC1 and PC2: (**a**) Factor loadings of selected variables (PV–peroxide value; K_232_, K_268_ –spectrophotometric indices; fatty acids C18:2 and C18:3; and volatile compounds) on the first two principal components (PC1 and PC2). (**b**) Score plot projecting the monovarietal virgin olive oils of Leccino (□), *Istarska bjelica* (ᴑ) and Buža (◊); unheated (green) and heated in an air oven for 1 h at 180 °C (blue) and 220 °C (red).

To better elucidate the parameters that distinguish monovarietal EVOOs unheated and heated at different heating temperatures, all samples were subjected to PCA. PCA was employed to distinguish samples based on volatile and phenolic compounds. By plotting the results in a two-dimensional space where the first two principal components (PC1 and PC2) represent the majority of data variability (69.3% in Figure 3 and 71.1% in Figure 4), the PCA allows the interpretation of the chemical changes occurring in monovarietal EVOOs during thermal exposure.

In Figure 3, the PCA projected the samples based on quality parameters and phenolic compounds. The results demonstrated a clear positive correlation between phenolic compounds (oleocanthal and hydroxytyrosol acetate) and oxidation indicators (PV, K_232_ and K_268_) (Figure 3a). This projection indicates that, as EVOO is heated, the relative concentration of these phenolic compounds changes in tandem with oxidation parameters. In particular, hydroxytyrosol acetate was highly correlated with the oxidation indicators (K_232_ and K_268_), confirming its increase during thermal oxidation and possible usage as a marker of oxidation. The score plot in Figure 3b displayed the samples along the x-axis (PC1), where the heating temperature clearly separated the samples. This separation highlights how heating promotes the degradation of phenolics, aligning fresh and thermally treated oils into distinct groups based on temperature. Further, a cultivar-specific response to heating emerged in this PCA, with B oils being distinguishable from L and IB oils based on their initial phenolic compositions, which influenced their degradation trajectories during heating. The noted results confirms the role of the initial phenolic composition related to the specific cultivar as a factor that influences changes during heating as previously reported [9].

In Figure 4, the PCA projected the samples based on quality parameters and volatile compounds. In the projection of samples, there was a clear separation according to the temperature of heating (Figure 4b), which indicated that the distribution of volatile compounds was primarily temperature-dependent. The projection of the selected variables in Figure 4a shows that most of the variables are located on the negative side of Factor 1, including (*E*)-2-hexenal and total volatile content (Figure 4a), being characteristic of the unheated EVOOs (Figure 4b). The PCA showed that oxidized volatile compounds (e.g., octanal, (*E*)-2-octenal, hexanal, 3-pentanone, and 1-penten-3-one) correlated positively with K_232_ and K_268_, being located on the positive side of PC1. This confirmed the role of these volatile compounds as thermal oxidation indicators. The cultivar was not a factor that led to the grouping of samples.

Unlike the phenolic composition analysis, the PCA of volatile compounds in Figure 4 reveals that cultivar type did not notably influence the grouping of samples. This observation suggests that while phenolic profiles can be cultivar-dependent, volatile degradation under thermal stress is more uniformly driven by temperature across different EVOO cultivars. The clear separation based on heating temperature affirms that oxidative changes in EVOO, particularly in volatile and phenolic profiles, are predominantly temperature-driven.

## 4. Conclusions

The results from this study indicated that the heating treatment influenced enhanced oxidative degradation in all the investigated monovarietal EVOOs, even though each oil cultivar showed some specificity during the course of the thermal degradation. Significant losses in oxidative stability and volatile compounds were observed after the heating treatment in each monovarietal olive oil. The decreases in the oxidative indicators (K_232_, K_268_, and ΔK) and phenolic and volatile compounds were more pronounced at a higher temperature of heating, which underlined the temperature dependency of the oxidative degradation during heating conditions. However, there was still a considerable amount of phenolic compounds and some signs of antioxidative activity preserved after the heating treatments. In addition to quality parameters that served as oxidation indicators, hydroxytyrosol acetate among phenolic compounds and octanal, (*E*)-2-octenal, hexanal, 3-pentanone, and 1-penten-3-one among the volatiles were underlined as possible thermal oxidation markers. Finally, under the investigated conditions, changes in the phenolic composition were cultivar-dependent aside from being influenced by the temperature of heating, while the content of volatile compounds distinguished samples primarily by the heating temperature with no significant influence of the specific cultivar. The obtained results contribute to the understanding of the fate and preservation of EVOO beneficial and flavor-related ingredients in the course of real domestic heating conditions and also to the understanding of changes occurring in the EVOO composition during exposure to different high temperatures as well as the role of the specific cultivar during heating.

## Figures and Tables

**Table 1:** Quality parameters (peroxide value—PV—and spectrophotometric indices—K_232_, K_268_, and ΔK) in monovarietal olive oil samples unheated and subjected to 1 h of heating at 180 and 220 °C.

	Leccino (L)			*Istarska bjelica* (IB)			Buža (B)			EVOO *
	Unheated	180 °C	220 °C	Unheated	180 °C	220 °C	Unheated	180 °C	220 °C	
PV (meq O_2_/kg)	5.07 ± 0.04 c	6.30 ± 0.05 b	8.25 ± 0.30 a	7.54 ± 0.07 a	6.89 ± 0.11 b	7.54 ± 0.03 a	8.23 ± 0.05 a	6.82 ± 0.16 c	7.61 ± 0.09 b	≤20.0
K_232_	1.66 ± 0.15 ab	1.23 ± 0.06 b	2.64 ± 0.82 a	2.12 ± 0.02 b	2.25 ± 0.06 b	3.30 ± 0.16 a	2.00 ± 0.04 c	2.28 ± 0.06 b	3.10 ± 0.05 a	≤2.50
K_268_	0.11 ± 0.00 c	0.35 ± 0.03 b	0.68 ± 0.05 a	0.18 ± 0.01 c	0.41 ± 0.06 b	0.71 ± 0.04 a	0.12 ± 0.00 c	0.40 ± 0.06 b	0.63 ± 0.07 a	≤0.22
∆K	0.00 ± 0.00 c	0.02 ± 0.00 b	0.04 ± 0.01 a	0.00 ± 0.00 c	0.01 ± 0.00 b	0.03 ± 0.01 a	0.00 ± 0.00 c	0.01 ± 0.00 b	0.03 ± 0.00 a	≤0.01

Data are expressed as means ± standard deviation (*n* = 3). Means within the single oil cultivar at different heating time labeled by different small letters show statistically significant differences between heating treatments (Tukey’s test, *p* ˂ 0.05). * Current limits for extra virgin olive oil (EVOO) category [25].

**Table 2:** The concentration of phenolic compounds in monovarietal olive oil samples unheated and subjected to 1 h of heating at 180 and 220 °C.

Phenolic Compound (mg/kg)	Leccino (L)			*Istarska bjelica* (IB)			Buža (B)		
	Unheated	180 °C	220 °C	Unheated	180 °C	220 °C	Unheated	180 °C	220 °C
Simple phenols									
Tyrosol	2.73 ± 0.13 a	2.73 ± 0.42 a	1.85 ± 0.14 b	3.58 ± 0.28 a	2.94 ± 0.02 b	2.47 ± 0.10 c	6.08 ± 0.76 a	5.71 ± 0.89 a	3.88 ± 0.40 b
Hydroxytyrosol	1.37 ± 0.14	1.55 ± 0.32	1.17 ± 0.05	1.93 ± 0.24 a	1.45 ± 0.02 b	1.25 ± 0.04 b	3.17 ± 0.39 a	2.84 ± 0.57 a	1.50 ± 0.14 b
Hydroxytyrosol acetate	0.29 ± 0.01 b	0.21 ± 0.03 b	0.39 ± 0.05 a	0.19 ± 0.02 c	0.35 ± 0.02 b	0.53 ± 0.06 a	0.26 ± 0.02 b	0.29 ± 0.02 b	0.62 ± 0.02 a
Vanillin	0.16 ± 0.01 a	0.11 ± 0.01 b	0.05 ± 0.01 c	0.16 ± 0.01 a	0.12 ± 0.00 b	0.06 ± 0.00 c	0.12 ± 0.01 a	0.08 ± 0.01 b	0.04 ± 0.00 c
Phenolic acids									
p-Coumaric acid	0.53 ± 0.02 a	0.48 ± 0.07 a	0.28 ± 0.04 b	1.63 ± 0.09 a	1.54 ± 0.03 a	0.92 ± 0.03 b	0.87 ± 0.07 a	0.76 ± 0.06 a	0.55 ± 0.07 b
Vanillic acid	0.36 ± 0.01 a	0.29 ± 0.04 a	0.14 ± 0.03 b	0.47 ± 0.02 a	0.42 ± 0.01 b	0.25 ± 0.00 c	0.43 ± 0.03 a	0.37 ± 0.02 b	0.26 ± 0.01 c
Flavonoids									
Luteolin	2.13 ± 0.16 a	1.70 ± 0.22 a	0.64 ± 0.20 b	2.63 ± 0.35 a	1.98 ± 0.12 b	0.81 ± 0.03 c	2.97 ± 0.5 a	2.13 ± 0.25 a	1.11 ± 0.25 b
Apigenin	0.40 ± 0.04 a	0.38 ± 0.05 a	0.20 ± 0.04 b	0.58 ± 0.09 a	0.51 ± 0.04 a	0.30 ± 0.03 b	0.75 ± 0.13 a	0.60 ± 0.08 ab	0.45 ± 0.09 b
Lignans									
Pinoresinol	3.45 ± 0.24	3.46 ± 0.35	3.36 ± 0.16	6.81 ± 0.57	6.64 ± 0.06	5.67 ± 0.60	4.53 ± 0.63	5.22 ± 0.53	4.83 ± 0.38
Acetoxypinoresinol *	6.91 ± 0.33 a	5.12 ± 0.57 b	2.78 ± 0.26 c	9.36 ± 0.63 a	6.64 ± 0.31 b	4.63 ± 0.49 c	10.9 ± 1.5 a	7.49 ± 0.63 b	5.94 ± 0.48 b
Secoiridoids									
Oleuropein + ligstroside aglycones I & II *	14.6 ± 0.4 a	5.91 ± 0.34 b	5.99 ± 0.55 b	22.2 ± 0.8 a	5.96 ± 0.55 b	2.05 ± 0.32 c	43.8 ± 5.5 a	11.1 ± 0.4 b	12.0 ± 0.2 b
Ligstroside aglycon (isomer II) *	9.71 ± 1.03 a	9.79 ± 1.41 a	4.20 ± 0.92 b	11.1 ± 2.1 a	8.57 ± 1.1 ab	6.27 ± 0.62 b	15.0 ± 3.1	15.5 ± 1.9	10.1 ± 2.0
Oleocanthal (p-HPEA-EDA) *	71.3 ± 1.6 ab	85.3 ± 12.8 a	59.4 ± 1.27 b	44.8 ± 2.7 b	73.0 ± 1.8 a	68.9 ± 7.2 a	60.2 ± 9.9 b	121 ± 14 a	146 ± 14 a
Oleuropein aglycone (isomer I) *	40.7 ± 0.4 a	14.3 ± 1.3 b	13.6 ± 0.3 b	66.7 ± 2.2 a	23.1 ± 0.2 b	15.1 ± 3.9 c	45.9 ± 2.3 a	15.3 ± 0.7 b	12.1 ± 0.5 b
Oleuropein aglycone (isomer II) *	22.3 ± 1.2 a	15.1 ± 1.2 b	4.46 ± 0.66 c	27.6 ± 2.2 a	11.1 ± 1.0 b	5.96 ± 0.84 c	27.9 ± 3.3 a	12.3 ± 0.7 b	6.04 ± 0.37 c
Oleuropein aglycone (isomer III) *	4.37 ± 0.29 a	4.82 ± 0.76 a	0.39 ± 0.2 b	4.70 ± 0.58 a	3.75 ± 0.40 a	0.55 ± 0.13 b	12.9 ± 2.0 a	9.39 ± 1.13 b	0.93 ± 0.31 c
Oleacein (3,4-DHPEA-EDA) *	113 ± 3 a	94.9 ± 15.0 a	55.2 ± 3.9 b	84.4 ± 3.3 b	111 ± 4 a	60.1 ± 2.1 c	72.4 ± 8.9 ab	85.4 ± 8.7 a	64.1 ± 6.4 b

Data are expressed as means ± standard deviation (*n* = 3). Means within the same row and single oil cultivar, at different heating time labeled by different small letters, are statistically different (Tukey´s test, *p* ˂ 0.05). * The phenolic compounds were quantified semi-quantitatively.

**Table 3:** The concentration of volatile compounds in monovarietal olive oil samples unheated and subjected to 1 h of heating at 180 and 220 °C.

Volatile Compound (mg/kg)	Leccino (L)			*Istarska bjelica* (IB)			Buža (B)		
	Unheated	180 °C	220 °C	Unheated	180 °C	220 °C	Unheated	180 °C	220 °C
C5 volatiles									
3-Pentanone	0.053 ± 0.000 c	0.115 ± 0.006 b	0.177 ± 0.011 a	0.045 ± 0.002 a	0.109 ± 0.008 c	0.200 ± 0.015 b	0.092 ± 0.002 b	0.111 ± 0.023 b	0.173 ± 0.013 a
1-Penten-3-one	0.013 ± 0.000 b	0.008 ± 0.000 c	0.016 ± 0.000 a	0.012 ± 0.001 b	0.010 ± 0.001 b	0.022 ± 0.002 a	0.012 ± 0.001 b	0.011 ± 0.001 b	0.017 ± 0.000 a
(E)-2-Penten-1-ol	0.023 ± 0.001 a	0.005 ± 0.001 b	0.002 ± 0.000 c	0.019 ± 0.001 a	0.005 ± 0.000 b	0.003 ± 0.000 c	0.024 ± 0.001 a	0.004 ± 0.001 b	0.002 ± 0.000 c
(E)-2-Pentenal	0.058 ± 0.001 a	0.037 ± 0.003 b	0.030 ± 0.002 c	0.049 ± 0.001 a	0.039 ± 0.002 b	0.040 ± 0.003 b	0.049 ± 0.001 a	0.041 ± 0.004 b	0.034 ± 0.002 c
Total C5 volatiles	0.147 ± 0.002 b	0.165 ± 0.006 b	0.225 ± 0.012 a	0.137 ± 0.018 b	0.162 ± 0.009 b	0.265 ± 0.017 a	0.177 ± 0.003 b	0.167 ± 0.029 b	0.225 ± 0.014 a
C6 volatiles									
Hexanal	1.52 ± 0.06 c	2.36 ± 0.23 b	3.61 ± 0.05 a	2.37 ± 0.14 b	2.65 ± 0.17 b	5.58 ± 0.23 a	2.34 ± 0.16 b	2.43 ± 0.55 b	4.34 ± 0.15 a
(E)-2-Hexenal	17.5 ± 0.1 a	1.04 ± 0.34 b	0.125 ± 0.034 c	21.4 ± 0.2 a	1.71 ± 0.28 b	0.232 ± 0.045 c	16.8 ± 0.1 a	0.763 ± 0.211 b	0.137 ± 0.025 c
(Z)-2-Hexenal *	0.112 ± 0.002 a	0.004 ± 0.002 b	0.002 ± 0.001 b	0.149 ± 0.005 a	0.008 ± 0.001 b	0.002 ± 0.000 b	0.094 ± 0.002 a	0.003 ± 0.001 b	0.000 ± 0.001 b
(E)-3-Hexenal *	0.077 ± 0.001 b	0.182 ± 0.043 a	0.156 ± 0.015 a	0.094 ± 0.001 b	0.221 ± 0.017 a	0.208 ± 0.021 a	0.061 ± 0.001 b	0.282 ± 0.049 a	0.241 ± 0.03 a
(Z)-3-Hexenal *	0.273 ± 0.000 a	0.013 ± 0.001 c	0.017 ± 0.001 b	0.468 ± 0.009 a	0.013 ± 0.001 b	0.017 ± 0.002 b	0.149 ± 0.009 a	0.011 ± 0.001 b	0.016 ± 0.001 b
Total C6 aldehydes	19.5 ± 0.2 a	3.61 ± 0.61 b	3.91 ± 0.01 b	24.5 ± 0.3 a	4.60 ± 0.37 c	6.04 ± 0.27 b	19.4 ± 0.2 a	3.49 ± 0.79 c	4.74 ± 0.20 b
1-Hexanol	1.09 ± 0.02 a	0.097 ± 0.010 b	0.061 ± 0.002 c	0.944 ± 0.007 a	0.120 ± 0.013 b	0.073 ± 0.007 c	1.54 ± 0.00 a	0.114 ± 0.025 b	0.066 ± 0.001 c
(E)-3-Hexen-1-ol	0.024 ± 0.000 a	0.007 ± 0.002 b	0.013 ± 0.007 b	0.026 ± 0.002 a	0.006 ± 0.001 b	0.009 ± 0.001 b	0.029 ± 0.004	0.014 ± 0.008	0.024 ± 0.029
(Z)-3-Hexen-1-ol	0.685 ± 0.006 a	0.059 ± 0.030 b	0.015 ± 0.001 b	1.14 ± 0.01 b	0.110 ± 0.020 c	0.019 ± 0.003 a	0.885 ± 0.007 a	0.047 ± 0.013 b	0.016 ± 0.001 c
(E)-2-Hexen-1-ol	0.804 ± 0.015 a	0.100 ± 0.013 b	0.093 ± 0.008 b	0.975 ± 0.006 a	0.123 ± 0.015 b	0.096 ± 0.019 b	1.609 ± 0.002 a	0.132 ± 0.054 b	0.079 ± 0.006 b
(Z)-2-Hexen-1-ol	0.009 ± 0.000 a	0.004 ± 0.002 b	0.005 ± 0.000 b	0.006 ± 0.000 b	0.005 ± 0.001 b	0.012 ± 0.003 a	0.010 ± 0.000 a	0.004 ± 0.001 b	0.009 ± 0.003 a
Total C6 alcohols	2.61± 0.04 a	0.267 ± 0.053 b	0.186 ± 0.017 b	3.09 ± 0.03 a	0.364 ± 0.043 b	0.210 ± 0.030 c	4.07 ± 0.01 a	0.311 ± 0.100 b	0.193 ± 0.034 b
Hexyl acetate	0.098 ± 0.004 a	0.062 ± 0.003 b	0.068 ± 0.002 b	0.069 ± 0.003 a	0.059 ± 0.003 b	0.067 ± 0.003 ab	0.353 ± 0.012 a	0.092 ± 0.014 b	0.076 ± 0.004 b
Total C6 volatiles	22.2 ± 0.2 a	3.94 ± 0.66 b	4.17 ± 0.12 b	27.7 ± 0.3 a	5.02 ± 0.40 c	6.31 ± 0.30 b	23.8 ± 0.2 a	3.89 ± 0.90 b	5.01 ± 0.24 b
Other									
(Z)-2-Penten-1-ol + (Z)-3-Hexenyl acetate	0.364 ± 0.001 a	0.015 ± 0.000 b	0.002 ± 0.000 c	0.360 ± 0.005 a	0.020 ± 0.003 b	0.002 ± 0.000 c	0.816 ± 0.013 a	0.046 ± 0.015 b	0.004 ± 0.001 c
3-Methylbutanal	0.009 ± 0.000 a	n.d. b	n.d. b	0.004 ± 0.000 a	0.001 ± 0.001 b	n.d. c	0.006 ± 0.000 a	0.002 ± 0.000 b	n.d. c
Octanal	0.088 ± 0.005 c	0.743 ± 0.094 b	1.70 ± 0.03 a	0.076 ± 0.012 c	0.589 ± 0.061 b	1.67 ± 0.18 a	0.089 ± 0.002 c	0.777 ± 0.239 b	1.76 ± 0.06 a
Ethyl 2-methylbutanoate	0.003 ± 0.001 b	0.007 ± 0.001 a	0.007 ± 0.000 a	0.003 ± 0.000 b	0.008 ± 0.002 a	0.009 ± 0.001 a	0.003 ± 0.001 b	0.011 ± 0.003 a	0.008 ± 0.001 a
Isoamyl acetate	0.006 ± 0.000	0.004 ± 0.001	0.004 ± 0.001	0.004 ± 0.001	0.003 ± 0.003	0.004 ± 0.001	0.007 ± 0.001 a	0.005 ± 0.001 b	0.003 ± 0.001 b
(E)-2-Octenal	0.066 ± 0.003 c	0.846 ± 0.082 b	1.33 ± 0.06 a	0.065 ± 0.001 c	0.869 ± 0.071 b	1.64 ± 0.18 a	0.064 ± 0.003 c	0.961 ± 0.220 b	1.52 ± 0.12 a
Acetic acid	0.220 ± 0.007 a	0.063 ± 0.001 c	0.120 ± 0.005 b	0.137 ± 0.002 a	0.067 ± 0.006 c	0.114 ± 0.009 b	0.182 ± 0.004 a	0.067 ± 0.011 c	0.104 ± 0.007 b
Total volatiles	23.1 ± 0.2 a	5.78 ± 0.64 c	7.56 ± 0.22 b	28.4 ± 0.3 a	6.74 ± 0.32 c	10.0 ± 0.7 b	25.2 ± 0.2 a	5.92 ± 1.38 c	8.63 ± 0.44 b

Data are expressed as means ± standard deviation (*n* = 3). Means within the same row and single oil cultivar at different heating time labeled by different small letters are statistically different (Tukey´s test, *p* ˂ 0.05). * The volatile compounds were quantified semi-quantitatively. n.d.—not detected.

## Data Availability

The original contributions presented in the study are included in the article. Further inquiries can be directed to the corresponding author.
